# Minimizing discordances in automated classification of fractionated electrograms in human persistent atrial fibrillation

**DOI:** 10.1007/s11517-016-1456-2

**Published:** 2016-02-25

**Authors:** Tiago P. Almeida, Gavin S. Chu, João L. Salinet, Frederique J. Vanheusden, Xin Li, Jiun H. Tuan, Peter J. Stafford, G. André Ng, Fernando S. Schlindwein

**Affiliations:** 1 Department of Engineering, University of Leicester, University Road, Leicester, LE1 7RH UK; 2 Department of Cardiovascular Science, University of Leicester, Leicester, UK; 3 University Hospitals of Leicester NHS Trust, Leicester, UK; 4 Biomedical Engineering, Engineering, Modelling and Applied Social Sciences Centre, Federal ABC University, Santo André, Brazil; 5 National Institute for Health Research Leicester Cardiovascular Biomedical Research Unit, Glenfield Hospital, Leicester, UK

**Keywords:** Atrial fibrillation, Substrate, Mapping, Electrogram fractionation, CFAE, Statistical classification

## Abstract

Ablation of persistent atrial fibrillation (persAF) targeting complex fractionated atrial electrograms (CFAEs) detected by automated algorithms has produced conflicting outcomes in previous electrophysiological studies. We hypothesize that the differences in these algorithms could lead to discordant CFAE classifications by the available mapping systems, giving rise to potential disparities in CFAE-guided ablation. This study reports the results of a head-to-head comparison of CFAE detection performed by NavX (St. Jude Medical) versus CARTO (Biosense Webster) on the same bipolar electrogram data (797 electrograms) from 18 persAF patients. We propose revised thresholds for both primary and complementary indices to minimize the differences in CFAE classification performed by either system. Using the default thresholds [NavX: CFE-Mean ≤ 120 ms; CARTO: ICL ≥ 7], NavX classified 70 % of the electrograms as CFAEs, while CARTO detected 36 % (Cohen’s kappa *κ* ≈ 0.3, *P* < 0.0001). Using revised thresholds found using receiver operating characteristic curves [NavX: CFE-Mean ≤ 84 ms, CFE-SD ≤ 47 ms; CARTO: ICL ≥ 4, ACI ≤ 82 ms, SCI ≤ 58 ms], NavX classified 45 %, while CARTO detected 42 % (*κ* ≈ 0.5, *P* < 0.0001). Our results show that CFAE target identification is dependent on the system and thresholds used by the electrophysiological study. The thresholds found in this work counterbalance the differences in automated CFAE classification performed by each system. This could facilitate comparisons of CFAE ablation outcomes guided by either NavX or CARTO in future works.

**Electronic supplementary material:**

The online version of this article (doi:10.1007/s11517-016-1456-2) contains supplementary material, which is available to authorized users.

## Introduction

Atrial fibrillation (AF) is the most common sustained arrhythmia in clinical practice and a leading cause of hospitalization and cardiovascular complications, particularly stroke. It is defined as a supraventricular tachyarrhythmia characterized by uncoordinated atrial activation with consequent deterioration of atrial mechanical function [4]. AF management consists of anticoagulation, antiarrhythmic drugs, electrical cardioversion, and radiofrequency catheter ablation [4]. The latter has been consolidated as the most accepted percutaneous procedure for AF treatment, achieving success rate as high as 90 % in patients with paroxysmal AF [4, 9]. Ablation is still suboptimal in patients with persistent or permanent atrial fibrillation (persAF) due to an incomplete understanding of the mechanistic interaction between relevant atrial substrate and the initiation and maintenance of AF.

**Fig. 1:**
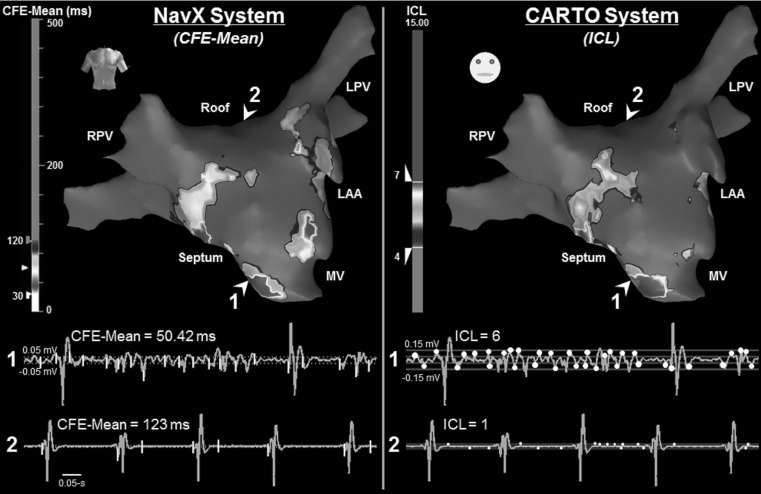
NavX (*left*) and CARTO (*right*) 3D atrial geometry representation for the same patient, with their respective automated CFAE detection algorithms. On the *bottom part* of the figure, the *top traces* refer to a segment of fractionated bipolar AEG (AEG 1), and *bottom traces* refer to a non-fractionated segment of bipolar AEG (AEG 2), both recorded from the LA endocardium. The AEG 1 has CFE-Mean = 50.42 ms and ICL = 6. The AEG 2 has CFE-Mean = 123 ms and ICL = 1. Explanation regarding the algorithms is provided in the text. *AEG* atrial electrogram, *CFE-Mean* index used by NavX to quantify AEG fractionation, *FI* fractionated interval, *ICL* interval confidence level: index used by CARTO to quantify AEG, *LPV* left pulmonary veins, *MV* mitral valve, *RPV* right pulmonary veins. These abbreviations were used in the subsequent figures

Sustained AF causes changes in the cardiac tissue characteristics, inducing structural and electric remodeling [6]. These regions can potentially host tissue with slow or inhomogeneous conduction, inducing reentry circuits, resulting in fractionated fibrillatory conduction [2], and are important in triggering and perpetuating atrial arrhythmias. Atrial electrograms (AEGs) acquired from such atrial substrate regions demonstrate the low amplitude, multiple deflection activations that characterize fractionated activity (Fig. 1). The ablation of atrial substrate hosting complex fractionated atrial electrograms (CFAEs) has been accepted by many as a useful additional therapy for persAF treatment [4]. Disparities in CFAE-guided ablation outcomes have, however, cast doubt on the efficacy of this approach (Table 1) [7, 14, 16, 19–21, 30, 31].

**Table 1:** Review of CFAE mapping systems, EGM settings, and success rate in different clinical studies

Study	EGM settings		Mapping system	No. patients	Success rate (%)
	Amplitude (mV)	Time (ms)			
Elayi et al. [7]	–	≤120	NavX	49	61
Lin et al. [14]	–	≤50	NavX	30	53
Nademanee et al. [16]	≤0.15	≤120	CARTO	121	95
Oral et al. [19]	–	≤120	CARTO	100	16
Oral et al. [20]	–	≤120	CARTO	50	18
Porter et al. [21]	0.05–0.15	60–120	CARTO	67	20
Verma et al. [30]	–	40–120	NavX	30	14
Verma et al. [31]	0.05≤	30–120	NavX	35	54

Previous studies conducted either additional or lone CFAE-guided ablation using different mapping systems and varying operator-defined settings, resulting in conflicting outcomes

Automated CFAE detection can be performed during electrophysiological studies by algorithms embedded in commercial three-dimensional electroanatomical mapping (3D EAM) systems [15, 31]. The two EAM systems being used in clinical practice for CFAE mapping are the NavX™ (St. Jude Medical, St. Paul, Minnesota) [31] and the CARTO (Biosense Webster, Diamond Bar, California) (Fig. 1) [15]. The algorithms embedded in those systems incorporate CFAE characteristics as initially described by Nademanee et al. [16]. Each algorithm, however, considers different premises to quantify fractionation, and the classification by the different systems does not always agree [13]. We hypothesized that the discordances between systems might result in different ablation target identification and generate discordant clinical results. In this study, we report a direct comparison of the automated CFAE classification performed by the algorithms embedded in NavX and CARTO. We also propose new thresholds for both primary and complementary indices to minimize the differences in CFAE classification performed by either system.

## Materials and methods

### Automated CFAE detection

NavX and CARTO provide primary indices to assess CFAE objectively, and complementary indices to further inform the electrophysiological study. Previous works have attempted to optimize CFAE detection using only the primary indices provided by the EAM systems [1]. There are currently no defined thresholds for the complementary indices to characterize CFAEs. Additionally, both systems allow for operator-defined settings—in this work referred to as “EGM settings”—to tune CFAE detection. Previous studies had attempted to optimize CFAE detection using these algorithms by varying EGM settings with different ablation outcomes (Table 1) [7, 14, 16, 19–21, 30, 31].

#### The NavX algorithm (EnSite System Version 8.0 Software, 2008)

NavX provides 3D EAM (Fig. 1 left) and online automated CFAE detection based on CFE-Mean. CFE-Mean is defined as the average fractionated interval (FI) between consecutive negative deflections (-dV/dt) inside a time window set by the user (from 1 to 8-s) of sequentially recorded bipolar AEGs (Fig. 1 bottom left) [31]. The negative deflections must meet three criteria in order to be marked: (1) exceed a peak-to-peak threshold greater than baseline noise; (2) have time duration within a threshold to avoid detection of ventricular far-field events; and (3) exceed a refractory period after the previous marked deflection to minimize multiple detections on a single deflection. NavX’s default EGM settings include a peak-to-peak sensitivity of 0.05 mV, deflection duration of less than 10 ms, and refractory period of 30 ms. AEGs with CFE-Mean within the range of 30–120 ms are considered to be fractionated [31]. These settings can be changed by the user (Table 1) [31]. NavX also computes the standard deviation of FI distribution inside a predefined time window as a complementary index, known as CFE-SD.

#### The CARTO algorithm (CARTO 3 System, 2008–2014, Version 4.3)

CARTO provides 3D EAM (Fig. 1 right) and online automated CFAE detection based on complex intervals occurring inside a 2.5-s window of sequentially recorded bipolar AEGs [15]. The algorithm identifies voltage peaks and troughs of bipolar AEGs that exceed a lower voltage threshold—to exclude noise—but do not exceed an upper voltage threshold. Similar to the NavX algorithm, the user can alter these CARTO thresholds (Table 1). The time intervals between successive peaks and troughs occurring within the voltage window are automatically marked by the system. The complex intervals marked within a time interval duration—defined by the operator—are identified during a 2.5-s time window (Fig. 1 bottom right). The number of identified complex intervals is referred to as the interval confidence level (ICL) and characterizes the repetitiveness of the CFAE complexes. CARTO’s default EGM settings consider a voltage window of 0.05–0.15 mV and a programmable time interval of 50–110 ms. Typically, ICL < 4 represents low fractionation, 4 ≤ ICL < 7 refers to moderate fractionation, and ICL ≥ 7 indicates high fractionation [15]. CARTO software also finds, as complementary indices, the average of the identified interval, referred to as the average complex interval (ACI), and the shortest identified interval, referred to as the shortest complex interval (SCI).

### Study population

The study population consisted of 18 persAF patients (16 male; mean age 56.1 ± 9.3 years; history of AF 67.2 ± 45.6 months) referred to our institution for first-time catheter ablation. Details of the clinical characteristics of the study subjects have been provided elsewhere [28]. Study approval was obtained from the local ethics committee, and all procedures were performed with full informed consent.

### Electrophysiological Study

Details of the electrophysiological study and mapping procedure have been described elsewhere [28]. Briefly, 3D left atrium (LA) geometry was created within [Ensite] NavX using a deflectable, variable loop circular PV mapping catheter (Inquiry Optima, St. Jude Medical). Pulmonary vein isolation (PVI) was performed, followed by the creation of a single roof line (Cool Path Duo irrigated RF catheter, St. Jude Medical). No additional ablation targeting CFAE was performed in this study. Sequential point-by-point bipolar AEGs were collected from 15 predetermined atrial regions before and after LA ablation with the ablation catheter [28]. A total of 797 AEGs were recorded from the LA, with a sampling frequency of 1.2 kHz, and band-pass filtered within 30–300 Hz. When an improvement of signal-to-noise ratio was necessary, a 50-Hz Notch filter was applied.

### Comparing CFAE definitions between EAM systems

#### Signal analysis

Each AEG, its corresponding CFE-Mean, and CFE-SD were exported from NavX with three time window lengths (2.5, 5, and 8-s). A validated off-line MATLAB algorithm was used to compute the ICL, ACI, and SCI of each exported AEG for CFAE identification as defined by CARTO—see Supplemental Materials.

Currently, the CARTO system considers only 2.5-s AEGs for CFAE detection. Hence, there is no validated ICL threshold for CFAE classification using time windows longer than 2.5 s. Nevertheless, the effects of different time windows—2.5, 5, 8 s—were assessed on ICL and CFE-Mean for the completeness of the investigation—see S*upplemental Materials*. Little influence on overall CFE-Mean was found when using different time windows. Therefore, for the remaining parts of the study, NavX and CARTO indices were measured using fixed 2.5-s AEG duration to allow a like-for-like comparison [15, 31].

#### Influence of EGM settings on CFAE classification

CFE-Mean and ICL were individually assessed, exploring the effects of varying EGM settings: NavX EGM settings (30–120 ms) and CARTO EGM settings (50–110 ms). Hence, the threshold for CFAE classification was 30–120 ms if CFE-Mean was measured using NavX EGM settings, and 50–110 ms for CARTO EGM settings [15, 31]. ICL ≥ 7 was used as the default threshold for CARTO CFAE categorization to assess the impact of both NavX and CARTO EGM settings [21].

#### CFAE detection thresholds for CFE-Mean and ICL

**Fig. 2:**
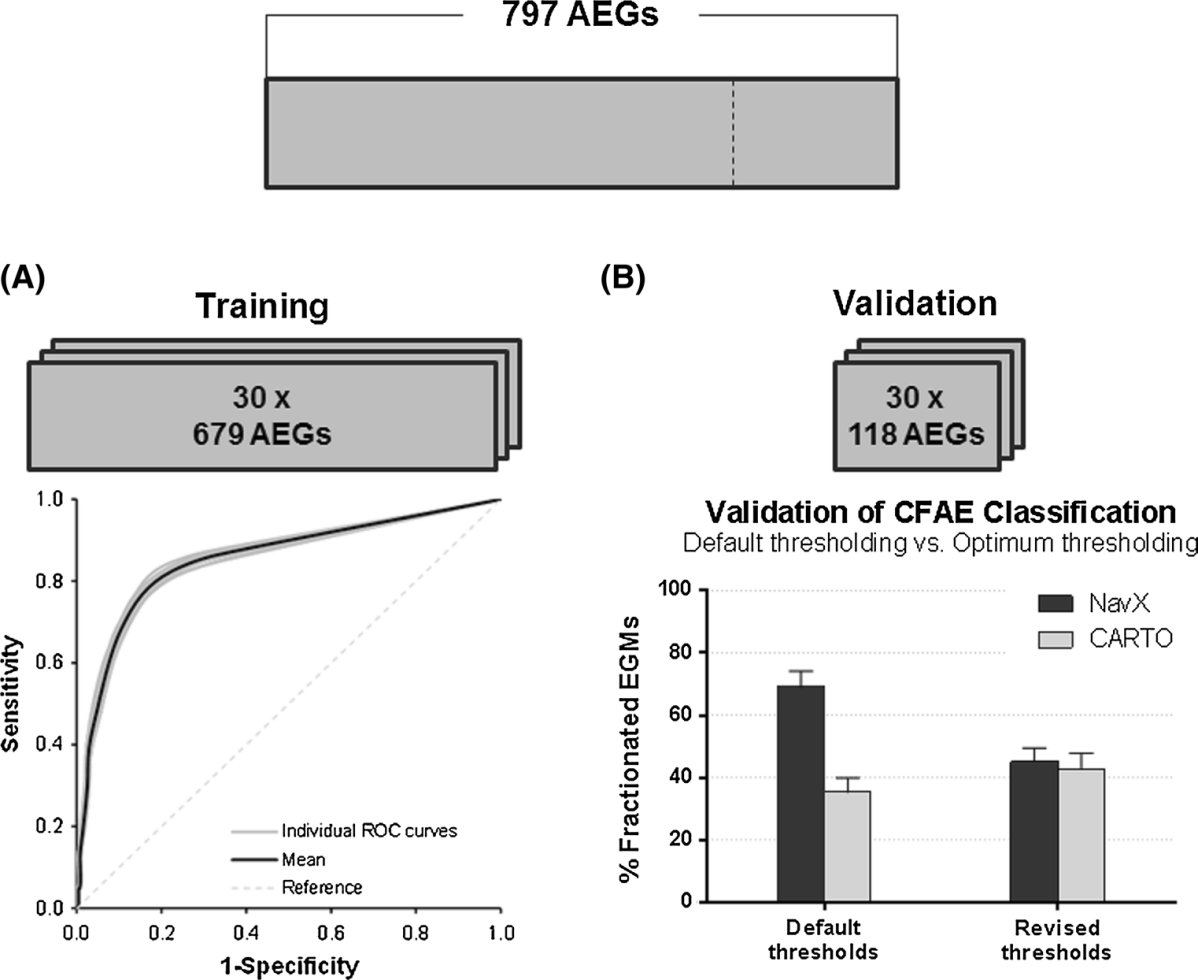
Illustration of the method for training the ROC curves and validating the proposed revised thresholds. **a** Thirty datasets—with 679 randomly selected AEGs each—were used to train and create the ROC curves. **b** For each of the 30 datasets, the remaining 118 AEGs were used to validate the thresholds found in the ROC curves

CFAE detection and classification were performed on 697 randomly sampled AEGs (out of the total 797), first using CFE-Mean and then ICL. This dataset was used to create receiver operating characteristic (ROC) curves and hence obtain the optimum sensitivity and specificity thresholds for both indices, using the counterpart index as the comparator (Fig. 2a) [8]. The ICL-based classification was assessed by creating a ROC curve, using CFE-Mean and the NavX EGM settings (CFE-Mean ≤ 120 ms) as the reference classification (CFAE/non-CFAE) [31]. The revised threshold for ICL was identified based on the optimum sensitivity and specificity on the ROC curve—defined as the point on the curve with the shortest distance to the top left corner of the graph. Similarly, the CFE-Mean-based classification was assessed by creating a ROC curve, using ICL and the CARTO EGM settings (ICL ≥ 7) as the reference classification (CFAE/non-CFAE) [21]. The revised threshold for CFE-Mean was identified based on the optimum sensitivity and specificity on the ROC curve. Area under the ROC curve (AUROC) and the *P* value were also calculated.

This process was iterated 30 times, each time with a different dataset of randomly sampled AEGs for ROC curve construction (697 AEGs), giving a total of 30 ROC curves for ICL and 30 for CFE-Mean in order to minimize data sampling and selection biasing.

#### CFAE detection thresholds for CFE-SD, ACI, and SCI

The revised thresholds for both CFE-Mean and ICL found in the ROC curves were used to perform a new CFAE classification on the 30 sets of 697 randomly sampled AEGs. In this new classification, an AEG was classified as CFAE only if both CFE-Mean and ICL agreed with the classification using their revised thresholds. These classifications were used to create ROC curves and hence obtain the optimum sensitivity and specificity thresholds for the complementary indices—CFE-SD, ACI, and SCI.

#### Validation of the revised thresholds for CFAE detection performed by NavX and CARTO

The revised thresholds found in the ROC curves for both NavX—CFE-Mean and CFE-SD—and CARTO—ICL, ACI, and SCI—were validated using the remaining 118 AEGs (30 sets of 118 AEGs randomly selected) (Fig. 2b).

For each of the 30 datasets, CFAE classification was performed using the combined assessment of both primary and complementary indices. Explicitly, an AEG was classified as CFAE if it complied with both CFE-Mean and CFE-SD for NavX classification. Similarly, an AEG was classified as CFAE if it complied with ICL, ACI, and SCI for CARTO classification.

### Statistical analysis

All continuous normally distributed variables are expressed as mean ± standard deviation (SD). Continuous non-normally distributed variables are expressed as median ± interquartile interval. Nonparametric paired multiple data were analyzed using the Friedman test with Dunn’s correction, while nonparametric unpaired data were analyzed using the Mann–Whitney test. Categorical data were expressed as percentages and analyzed using the two-sided Yates-corrected Chi-square test. The quantification of the agreement between the rankings made by CFE-Mean and ICL—as measured using their default settings—was assessed by the Spearman’s correlation between both indices. The level of agreement in the CFAE classification performed by the two systems was assessed by the Cohen’s kappa (*κ*) score [5]. Kappa score within range 0 ≤ *κ* < 0.4 suggests marginal agreement between two indices; 0.4 ≤ *κ* ≤ 0.75 good agreement; and *κ* > 0.75 excellent agreement [12]. *P* values less than 0.05 were considered statistically significant.

## Results

### Influence of EGM settings on CFAE classification

CFAE classification differed as performed by CFE-Mean and ICL using their respective default EGM settings. CFE-Mean classified 70 % of the AEGs as CFAEs using NavX EGM settings, while ICL classified 36 % using CARTO EGM settings (*P* < 0.0001).

**Fig. 3:**
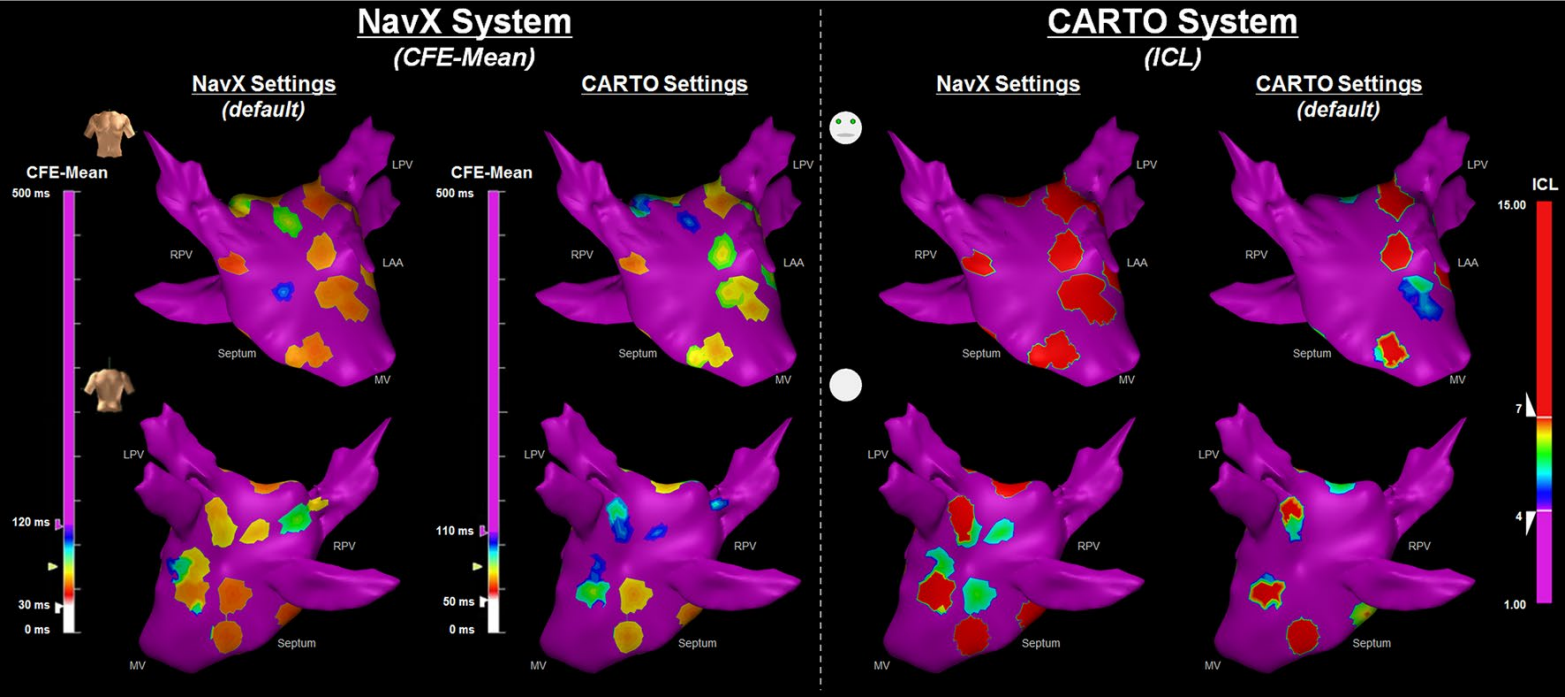
3D CFAE map showing the anterior (*upper*) and posterior (*bottom*) view of the LA from one patient according to CFE-Mean (*left-hand side*) and ICL (*right-hand side*), using different EGM settings. NavX EGM settings identify more regions of the LA as CFAE than CARTO’s when applied to either CFE-Mean or ICL

Changing the EGM settings alters CFAE classification. Figure 3 demonstrates the importance of EGM settings for CFAE classification. It illustrates the 3D CFAE map (anterior and posterior views) of one of the patients according to CFE-Mean and ICL, using different EGM settings. CFE-Mean measured using NavX’s settings identified more atrial regions as CFAEs, while ICL measured using CARTO’s settings showed fewer regions as fractionated. When analyzing the entire database, the NavX EGM settings consistently categorized more AEGs as fractionated than the CARTO EGM settings (70 vs. 54 %, *P* < 0.0001 for CFE-Mean; 62 vs. 36 %, *P* < 0.0001 for ICL).

### CFAE detection thresholds for CFE-Mean and ICL

**Fig. 4:**
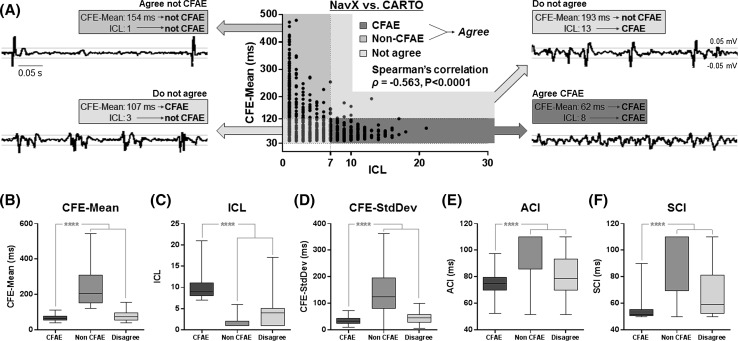
**a** Comparison between CFE-Mean and ICL measured for all 797 AEGs, as determined by default NavX and CARTO EGM settings, respectively. Their respective default thresholds are highlighted (CFE-Mean ≤ 120 ms; ICL ≥ 7). Four quadrants were delimited: two quadrants where ICL and CFE-Mean agreed in terms of categorization, i.e., whether an AEG is fractionated or not fractionated, and two quadrants in which they disagreed. Examples of AEGs for each of the quadrants are shown, to illustrate the characteristics of each group. 230 (out of 797) AEGs with organized activations were found in the non-fractionated agreement zone (*green*). When looking at the AEGs corresponding to the disagreement quadrants (*gray*), one notices that they are less organized, still with distinguishable activations. In one *gray region* (*bottom left*), 282 AEGs have been classified as CFAEs by NavX but not by CARTO. In the other *gray region* (*top right*), 12 AEGs have been classified as CFAEs by CARTO, but not by NavX. Finally, 273 highly fractionated AEGs were found in the CFAE agreement zone (*red*). The distributions for the AEGs classified as non-CFAEs or CFAEs by both systems, as well as AEGs that had different classifications for each system, are shown according to CFE-Mean (**b**), ICL (**c**), CFE-SD (**d**), ACI (**e**), and SCI (**f**). ****P < 0.0001

The comparison between CFE-Mean and ICL (measured using their default settings) for each of the 797 AEGs is illustrated in Fig. 4a. The respective default thresholds for both CFAE detection techniques are highlighted. The four quadrants delimited by the thresholds illustrate the zones of agreement and disagreement between CFE-Mean and ICL. Spearman’s correlation between the classifications by the two indices was *ρ* = −0.563 (*P* < 0.0001). 230 (out of 797) AEGs with organized activations were found in the non-fractionated agreement zone (green). When looking at the AEGs corresponding to the disagreement quadrants (gray), 282 AEGs have been classified as CFAEs by NavX but not by CARTO in one gray region (bottom left). In the other gray region (top right), 12 AEGs have been classified as CFAEs by CARTO, but not by NavX. Finally, 273 highly fractionated AEGs were found in the CFAE agreement zone (red).

Quantitative results are provided in Fig. 4b–f to characterize the AEGs after the objective comparison between CFE-Mean and ICL. The results show the distributions for primary and complementary indices (CFE-Mean, ICL, CFE-SD, ACI, and SCI, respectively) for the AEGs classified as non-CFAEs or CFAEs by both systems, as well as AEGs that had different classifications for each system. For all indices, the distributions for the AEGs classified as CFAE (red) were significantly different (*P* > 0.0001) than the combined distributions of non-CFAEs together with those AEGs with different classifications for each system (green + gray).

**Fig. 5:**
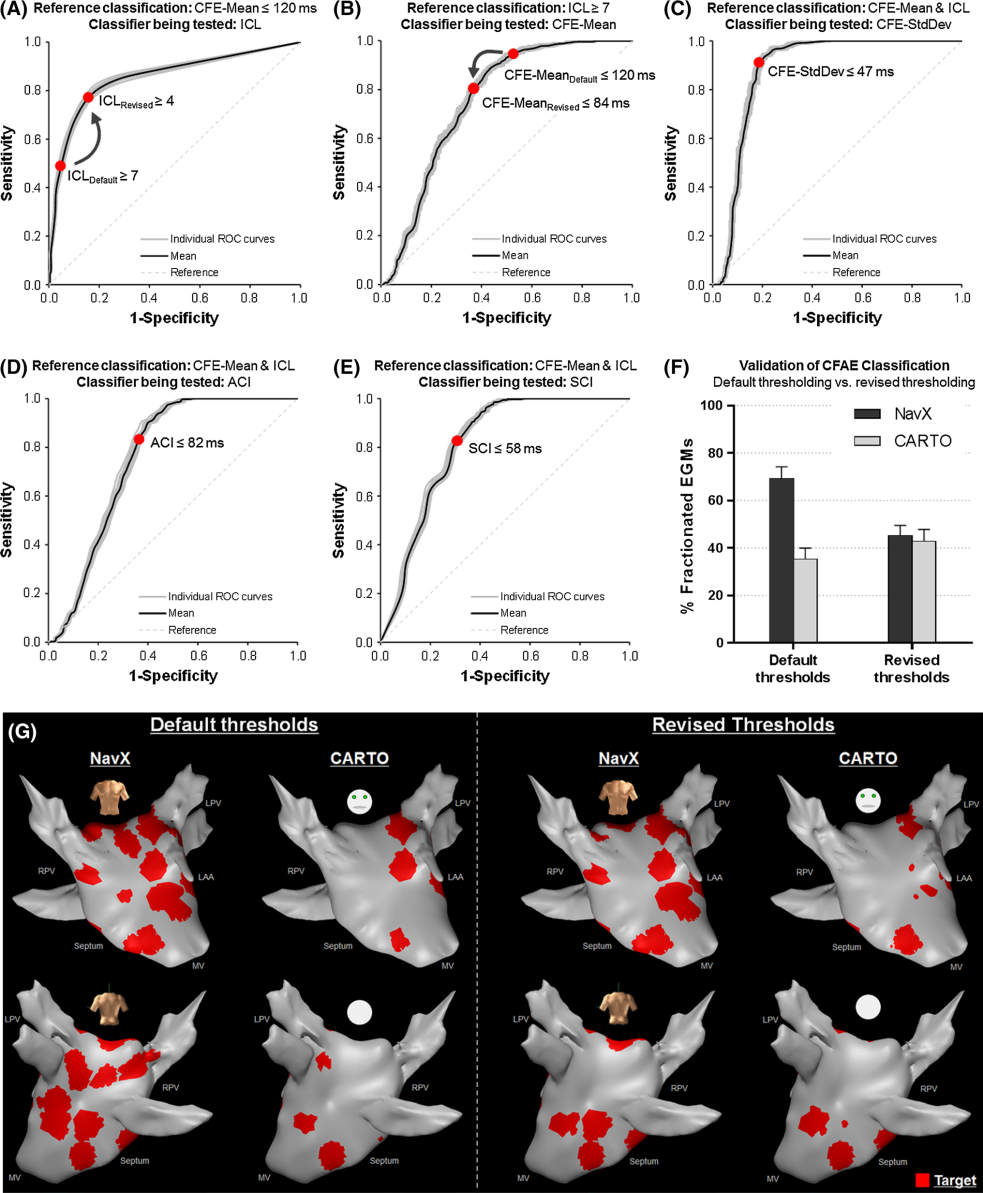
Mean (*black lines*) and individual (*gray lines*) receiver operating characteristic (ROC) curves used to adjust CFAE classification. ROC curve of **a** ICL-based classification using CFE-Mean ≤ 120 ms as the reference classification and; **b** CFE-Mean-based classification using ICL ≥ 7 as the reference classification. The CFAE classification in agreement with CFE-Mean and ICL using their revised thresholds (CFE-Mean ≤ 84.1 ± 0.4 ms; ICL ≥ 3.8 ± 0.4) was used to create ROC curves of **c**–**e**. **c** CFE-SD; **d** ACI and; **e** SCI. The area under the ROC curve (AUROC) and optimum sensitivity and specificity for each measure are listed in Tables 2 and 3. **f** Validation of the revised thresholds for CFAE classification. **g** Illustration of CFAE classification performed by NavX and CARTO in the LA from one patient using their default (*left*) and revised (*right*) thresholds. Explanations are provided in the text

Figure 5a, b shows the ROC curves according to the CFAE classification performed by CFE-Mean and ICL. Table 2 provides the sensitivity, specificity, and AUROC values for each case. The details of the ROC curves from the 30 datasets are provided in the Supplemental Materials.

**Table 2:** Sensitivity and specificity for A ICL-based classification using CFE-Mean ≤ 120 ms as the reference classification, and B CFE-Mean-based classification using ICL ≥ 7 as the reference classification

	Reference classifier	Classifier being tested	Sensitivity	1-Specificity	AUROC	*P* value
A	CFE-Mean ≤ 120 ms	ICL_Default_ ≥ 7	0.492 ± 0.008	0.050 ± 0.005	0.852 ± 0.005	****
		ICL_Revised_ ≥ 3.8 ± 0.4	0.777 ± 0.022	0.162 ± 0.022		
B	ICL ≥ 7	CFE-Mean_Default_ ≤ 120 ms	0.958 ± 0.005	0.552 ± 0.009	0.755 ± 0.005	****
		CFE-Mean_Revised_ ≤ 84.1 ± 0.4 ms	0.807 ± 0.010	0.362 ± 0.006		

Mean (± SD) of each sensitivity/specificity point from the 30 receiver operating characteristic (ROC) curves according to the CFAE classification performed by CFE-Mean and ICL. The mean (± SD) area under the ROC curve (AUROC) and optimum sensitivity and specificity for each measure are listed

*AUROC* area under receiver operating characteristic curve, *ICL* interval confidence level

**** *P* < 0.0001

The ICL-based classification suggests that the default threshold for CARTO (ICL ≥ 7) provides high specificity but poor sensitivity for CFAE detection (Fig. 5a; Table 2A). The revised threshold found from the ROC curves (ICL ≥ 3.8 ± 0.4) provides optimum sensitivity and specificity for CFAE detection and classification using CFE-Mean ≤ 120 ms as the reference classification.

The CFE-Mean-based classification suggests that the default threshold for NavX (CFE-Mean ≤ 120 ms) provides high sensitivity but poor specificity for CFAE detection (Fig. 5b; Table 2B). The revised threshold found from the ROC curves (CFE-Mean ≤ 84 ± 0.4 ms) provides optimum sensitivity and specificity for CFAE detection and classification using ICL ≥ 7 as the reference classification.

Other thresholds for CFE-Mean- and ICL-based classification have been explored and support the present findings. Details are provided in the Supplemental Materials.

### CFAE detection thresholds for CFE-SD, ACI, and SCI

Figure 5c–e shows the ROC curves for CFE-SD, ACI, and SCI according to the CFAE classification performed by both CFE-Mean and ICL, using their revised thresholds. Table 3 provides the sensitivity, specificity, and AUROC values. The details of the ROC curves from the 30 datasets are also provided in the Supplemental Materials.

**Table 3:** Sensitivity and specificity for CFE-SD, ACI, and SCI according to CFAE classification agreement between CFE-Mean and ICL

Thresholds	Sensitivity	1-Specificity	AUROC	*P* value
CFE-SD ≤ 46.6 ± 0.8 ms	0.905 ± 0.012	0.185 ± 0.008	0.877 ± 0.014	****
ACI ≤ 82.2 ± 0.3 ms	0.827 ± 0.010	0.360 ± 0.009	0.759 ± 0.006	****
SCI ≤ 58.6 ± 0.4 ms	0.816 ± 0.012	0.300 ± 0.009	0.812 ± 0.005	****

Mean (± SD) of each sensitivity/specificity point from the 30 receiver operating characteristic (ROC) curves according to the CFAE classification performed concurrently by both CFE-Mean and ICL. The mean (± SD) area under the ROC curve (AUROC) and optimum sensitivity and specificity for each measure are listed. **** *P* < 0.0001

The ROC curves suggest that CFE-SD ≤ 46.6 ± 0.8 ms (Fig. 5c), ACI ≤ 82.2 ± 0.3 ms (Fig. 5d), and SCI ≤ 58.6 ± 0.4 ms (Fig. 5e) provide optimum sensitivity and specificity for CFAE detection, when considering the agreement between CFE-Mean and ICL for CFAE classification.

### Validation of the revised thresholds for CFAE detection performed by NavX and CARTO

Using the default thresholds (NavX: CFE-Mean ≤ 120 ms; CARTO: ICL ≥ 7), NavX classified 69 ± 5 % of the AEGs from the internal validation datasets as CFAEs, while CARTO detected 35 ± 5 % (*P* < 0.0001). With the revised thresholds (NavX: CFE-Mean ≤ 84.1 ± 0.4 ms and CFE-SD ≤ 46.6 ± 0.8 ms; CARTO: ICL ≥ 3.8 ± 0.4, ACI ≤ 82.2 ± 0.3 ms and SCI ≤ 58.6 ± 0.4 ms), NavX classified 45 ± 4 %, while CARTO detected 42 ± 5 % (*P* < 0.0001). These results are illustrated in Fig. 5f.

Figure 5g illustrates CFAE classification performed by NavX and CARTO using the default (left-hand side) and revised (right-hand side) thresholds for the same patient. The CFAE maps produced by both systems using their default thresholds are very discordant, and these differences were minimized when each system used their revised thresholds. The CFAE maps created by both systems using their revised thresholds identified more similar atrial regions as target for ablation. The Kappa score between the CFAE categorization performed by NavX and CARTO significantly increased (*P* < 0.0001) from *κ* = 0.34 ± 0.07 (marginal agreement, *P* < 0.0001) using their default thresholds to *κ* = 0.45 ± 0.10 (good agreement, *P* < 0.0001) with the proposed revised thresholds.

## Discussion

This is the first study that uses the same bipolar AEG data collected during persAF ablation to compare CFAE detection performed by the algorithms embedded in NavX and CARTO systems. Additionally, the thresholds for the indices used by both systems were adjusted to minimize the differences between them. The results presented here highlight the discordances in CFAE classification between both systems, which could produce potential disparities in CFAE-guided ablation. The proposed revised thresholds counterbalance the differences in automated CFAE classification performed by the algorithms embedded in each system and reduce the discordances between them. Unifying methods of CFAE classification would allow comparable CFAE maps to be generated which could then act as a standard for future clinical studies.

### Atrial substrate characterized by CFAE

The true significance of CFAE in the pathophysiology of AF remains to be determined. Although it is believed that CFAEs represent atrial substrate during persAF [2, 4, 16, 23, 32], recent investigations have shown that fractionated AEGs during AF may characterize remote atrial far-field activity [3, 17] and passive wavefront collision within the atrial anatomy [24]. Recent work has reported no benefit when ablation of CFAEs was performed in addition to PVI in persAF patients [29]. However, this work has been openly criticized for (1) not considering the combination of PVI, anatomical lines creation and CFAE ablation, and (2) the CFAE mapping algorithm used [26]. Discordances in CFAE mapping algorithms create significant difficulty in comparing CFAE ablation studies. Minimizing the differences in CFAE classification between NavX and CARTO may help to clarify the significance of CFAE as a driver of persAF. Therefore, prudence is needed when comparing the outcomes in AF ablation incorporating CFAE-targeted approaches in different electrophysiological studies using different mapping systems [7, 14, 16, 19–21, 29–31].

### The lack of a gold standard for CFAE definition

There is currently no gold standard for CFAE classification in human persAF. This remains one of the biggest challenges for CFAE-based ablation. Visual assessment performed by experts could help on arbitration of fractionation, but would also introduce subjectivity to the method as each specialist has his/her own perception of what defines fractionation [1, 10, 18]. Additionally, even if the experts were able to visually identify if an AEG is a CFAE, still this would not be sufficient to effectively conclude that the AEG is a true representation of atrial substrate (these would correspond to atrial regions that organize AF when ablated). This ultimate conclusion is only possible by assessing if AF becomes more organized after ablating this particular atrial region by assessing if there is an appreciable change in the rhythm—either AF termination or increase in AF cycle length. Any “external reference” other than “AEGs that organized AF after ablation” would only introduce subjectivity to the method and would contribute little to objectively identifying LA regions as a surrogate of pro-arrhythmogenic sites. Therefore, visual assessment of fractionation for the recorded signals performed by clinicians was not included in this study. The present study focused on the classification performed automatically by both systems as currently performed in CFAE-guided ablation therapy, without the interference and subjectivity induced by operators. This allowed for an objective investigation of CFAE detection by investigating each system—one at a time—as being the “gold” standard for CFAE classification.

### CFAE detection performed by NavX and CARTO algorithms

We have shown that CFAE target identification is dependent on the system used and settings applied during the procedure. Different CFAE mapping algorithms are based on different premises to measure fractionation. For instance, NavX identifies AEGs with a very short cycle length with or without multiple potentials. CARTO, on the other hand, mostly measures AEGs that are composed of two deflections or more and/or have a perturbation of the baseline with continuous deflections [16].

The comparison between both mapping systems using their default settings produced a low correlation (*ρ* = −0.563, *P* < 0.0001), which supports recently published data [13]. However, the Spearman’s correlation does not consider the thresholds for CFAE classification. The values—and correlation—of CFE-Mean and ICL only have a full electrophysiological meaning when linked with the thresholds used. This information is provided by the Kappa score.

It is known that both systems are not used simultaneously during atrial substrate mapping and that physicians frequently vary the settings for CFAE mapping in a patient-specific manner [7, 14, 16, 19–21, 29–31]. Our results propose revised thresholds for CFAE detection to be used independently by NavX and CARTO to even out the discordances between them. Therefore, a CFAE map created with NavX utilizing the revised NavX’s thresholds will look more similar to the one that would have been created with CARTO utilizing CARTO’s revised thresholds proposed in this work, as illustrated in Fig. 5g.

The use of both primary and complementary indices is an additional way to even out differences in CFAE classification performed by each system. There are little data available about the complementary indices measured by automated algorithms being used to either target or support CFAE identification during atrial substrate mapping [22, 25, 27]. However, the ROC curves generated using the agreement between CFE-Mean and ICL as the reference for the classification of CFE-SD, ACI, and SCI (Fig. 5c–e) provide evidence that these complementary indices can help to distinguish CFAE from non-CFAE effectively. This would further improve the agreement of CFAE classification performed by both systems.

## Limitations

The current study was limited to retrospective data. Further understanding of the underlying cardiac electrophysiological mechanisms behind CFAEs would be helpful for the validation of the suggested revised thresholds, such as in (1) computational intracardiac models that simulate both atrial electrical activity and ablation procedures during AF [11], and (2) prospective studies using the revised thresholds in the identification of ablation targets during substrate mapping.

## Conclusions

This study provides a direct quantitative comparison of CFAE detection during persAF, applying the automated algorithms embedded in NavX and CARTO systems to the same bipolar AEG data. We have demonstrated that CFAE mapping (and thus ablation target identification) varies significantly for the same individual, depending on the system and its settings. Our work takes a first step to understanding and minimizing the discordance between NavX and CARTO. We propose revised thresholds that adjust sensitivity and specificity of CFAE detection as independently performed by NavX (CFE-Mean ≤ 84 ms; CFE-SD ≤ 47 ms) and CARTO (ICL ≥ 4; ACI ≤ 82 ms; SCI ≤ 58 ms). These thresholds counterbalance the intrinsic differences between the CFAE algorithms embedded in each system, allowing comparable CFAE maps to be generated which would facilitate the direct comparison of CFAE-guided ablation outcomes in future studies.

## Electronic supplementary material

Below is the link to the electronic supplementary material.

Supplementary material 1 (DOC 646 kb)
