# Supplementary material for: Minimizing discordances in automated classification of fractionated electrograms in human persistent atrial fibrillation
Source: Med Biol Eng Comput. 2016 Feb 25;54(11):1695–706. doi: 10.1007/s11517-016-1456-2 (PMC5069340; doi:10.1007/s11517-016-1456-2)
Supplement: Supplementary file 1 — Supplementary material 1 (DOC 646 kb) [file 11517_2016_1456_MOESM1_ESM.doc]

**Minimizing discordances in automated classification of fractionated electrograms in human persistent atrial fibrillation**

**Supplemental Material***

***intended for publication as an online data supplement**

Tiago P Almeida, MSc*; Gavin S Chu, MB BChir, MA(Cantab), MRCP(UK)‡§; João L Salinet, PhD*†; Frederique J Vanheusden, PhD*; Xin Li, MSc*; Jiun H Tuan, MBChB, MD, MRCP§; Peter J Stafford, MB BS, MD, FRCP§; G André Ng, MBChB, PhD, FRCP(Glasg), FRCP, FESC‡§#; Fernando S Schlindwein, PhD, DSc*#

*Department of Engineering, University of Leicester, UK; ‡Department of Cardiovascular Science, University of Leicester, UK; §University Hospitals of Leicester NHS Trust, UK;† Biomedical Engineering, Engineering, Modelling and Applied Social Sciences Centre, Federal ABC University, Brazil; #National Institute for Health Research Leicester Cardiovascular Biomedical Research Unit, Glenfield Hospital, UK.

**Address for correspondence:**

Dr. Fernando S. Schlindwein,

Department of Engineering, University of Leicester,

University Road, Leicester, LE1 7RH, England, UK

Email: f.s.schlindwein@leicester.ac.uk

Tel: +44 (0)116 252 5053

Fax: +44 (0)116 252 2619

**Validation of an algorithm to reproduce CARTO CFAE definition**

***Introduction***

CARTO software provides 3D EAM and online automated CFAE detection based on selected voltage peaks and troughs inside a 2.5-s window of sequentially recorded bipolar EGMs. The algorithm subsequently identifies voltage peaks and troughs of bipolar EGMs that exceed a user defined programmable lower voltage threshold to exclude noise, but that do not exceed an upper voltage threshold. The time intervals between successive peaks and/or troughs falling within the voltage window are measured. Complex intervals that fall within a user defined programmable duration are identified during the entire 2.5-s time window (Figure 1B). The number of identified complex intervals is referred to as Interval Confidence Level (ICL), and characterizes the repetitiveness of the CFAE complexes. The shortest identified interval is referred to as Shortest Complex Interval (SCI), while the average is referred to as Average Complex Interval (ACI). The default CARTO EGM settings consider a voltage window of 0.05-0.15 mV and a programmable time interval of 50-110 ms (Table 1). Typically, ICL < 4 represents low fractionation, 4 ≤ ICL < 7 refers to moderate fractionation and ICL ≥ 7 indicates high fractionation.

The objective of this part of the study was to validate a MATLAB® algorithm that reproduces CARTO CFAE definition and computes ICL, SCI and ACI.

***Methods***

587 sequentially recorded EGMs were exported from 3 patients referred to our institution for catheter ablation of persAF guided by CARTO. All EGMs were recorded with sampling frequency of 1 kHz, and filtered with a 30–300 Hz band-pass filter. Signals were exported with 2.5-s recording duration using CARTO EGM settings (0.05-0.15 mV; 50-110 ms). The peaks and troughs of an EGM were identified. Those that exceeded a user defined programmable lower voltage threshold to exclude noise, but that did not exceed an upper voltage threshold, were marked. The time intervals between successive marked peaks and/or troughs falling within the voltage window were measured. ICL, SCI and ACI were identified within the recorded 2.5-s EGM. The ICL, SCI and ACI computed by the MATLAB script were compared directly with CARTO system output.

*Statistical Analysis*

All continuous non-normally distributed variables are expressed as median ± interquartile interval. Nonparametric unpaired data were analyzed by Mann–Whitney test. The level of statistical significance was set at a P<0.05.

***Results***

Figure S1 illustrates the comparison between the ICL, ACI and SCI measured by both CARTO and the developed MATLAB algorithm. Agreement of ICL, ACI and SCI measured using CARTO and the MATLAB algorithm was 100%.

**Figure S1**

**Figure S1.** Comparison between ICL, ACI and SCI as computed by CARTO system and the MATLAB algorithm. **A.i.** ICL. **A.ii.** ICL correlation between CARTO and MATLAB. Agreement between CARTO and MATLAB was 100%. **B.i.** ACI. **B.ii**. ACI correlation between CARTO and MATLAB. Agreement between CARTO and MATLAB was 100%. **C.i.** SCI. **C.ii.** SCI correlation between CARTO and MATLAB. Agreement between CARTO and MATLAB was 100%.

**Influence of AEG duration on ICL and CFE-Mean**

***Methods***

Currently, the CARTO system considers only AEGs with 2.5-s duration for CFAE detection using ICL. Hence, there is no validated fractionation threshold for ICL using time windows longer than 2.5-s. Nevertheless, the effects of different time windows – 2.5-s, 5-s, 8-s – were assessed on overall ICL and CFE-Mean for the completeness of the investigation.

797 AEGs were and its corresponding CFE-Mean was exported from NavX with three time window lengths (2.5-s, 5-s and 8-s). The default threshold for fractionation detection was 30-120 ms if CFE-Mean was measured using NavX EGM settings and 50-110 ms for CARTO EGM settings. The validated offline algorithm was used to compute the ICL of each exported AEG for CFAE identification as defined by CARTO. ICL ≥ 7 was used as the default threshold for ICL CFAE categorization.

***Results***

The results suggest that there was no significant difference between CFE-Mean measured within 2.5-s vs. 5-s vs. 8-s AEGs segments, using either NavX EGM settings or CARTO EGM settings (P≈1.00 for 2.5-s vs. 5-s vs. 8-s using NavX EGM settings; P≈1.00 for 2.5-s vs. 5-s and 5-s vs. 8-s, and P=0.2443 for 2.5-s vs. 8-s using CARTO EGM settings). On the other hand, longer AEG duration increased ICL irrespective of EGM settings (P<0.0001 for all cases).

We observed that different time windows had little influence on overall CFE-Mean. In contrast, longer time windows significantly increased ICL, as expected. As ICL is the *count* of complex intervals within a time interval, it can be expected that longer time window lengths would have higher ICL. There is currently no validated fractionation threshold for ICL using time windows longer than 2.5-s. The default settings for ICL in CARTO considers windows of 2.5-s. On the other hand, NavX allows for different time window lengths during CFAE analysis (from 1-s to 8-s). As CFE-Mean is the *average* of the FIs within a given time window, it can be inferred that longer time window lengths would not affect CFE-Mean significantly.

Since our data showed that different AEG durations had little influence on CFE-Mean, NavX and CARTO algorithms could be compared using the same AEG data with a fixed AEG duration of 2.5-s, following the default settings for ICL in CARTO.

**CFAE detection thresholds for CFE-Mean and ICL**

***Methods***

Receiver operating characteristic (ROC) curves were created to find optimum thresholds for ICL and CFE-Mean according to the CFAE classifications performed by each opposite metric. Firstly, CFAE classification (CFAE / non-CFAE) was performed on all 797 AEGs by a fixed CFE-Mean threshold. Eight CFE-Mean thresholds were considered (CFE-Mean ≤ 120; 110; 100; 90; 80; 70; 60; 50 ms). Each classification was then used to create ROC curves by varying ICL. Individual optimal values for ICL were found based on the optimum sensitivity and specificity of each ROC curve. Similarly, CFAE classification (CFAE / non-CFAE) was performed on all 797 AEGs by a fixed ICL threshold. Four ICL thresholds were considered (ICL ≥ 4; 5; 6; 7). Each classification was then used to create ROC curves by varying CFE-Mean. Individual optimal values for CFE-Mean were found based on the optimum sensitivity and specificity of each ROC curve.

Additionally, approximately 90% (697) of the total AEGs were randomly selected to create ROC curves using only the default thresholds for CFE-Mean and ICL. The remaining 10% (100) AEGs were used to validate and compare the CFAE classification performed by CFE-Mean and ICL using both default and optimum thresholds found in the ROC curves. This process was repeated thirty times, each time with a different set of 90%/10% randomly selected AEGs. Therefore, thirty ROC curves were created for ICL and thirty for CFE-Mean.

This part of the study considered AEGs with 2.5-s, ICL measured with CARTO EGM settings and CFE-Mean measured with NavX EGM settings. The area under the ROC curve (AUROC) and optimum sensitivity and specificity were calculated for each measure.

***Results***

Figure S2A shows the ROC curve according to the CFAE classification performed by CFE-Mean (CFE-Mean ≤ 120; 110; 100; 90; 80; 70; 60; 50 ms) and varying ICL. Table S1 shows the AUROC, the default and optimum threshold for ICL, alongside the sensitivity and specificity found on the ROC curves for both thresholds. The results from the ROC curve suggest that the default threshold for CARTO (ICL ≥ 7) provided a high specificity, but poor sensitivity for CFAE detection in all cases. The proposed threshold (ICL ≥ 4) provides the optimum sensitivity and specificity for CFAE detection in accordance to the CFAE classification performed by NavX.

Figure S2B shows the ROC curve according to CFAE classification performed by ICL (ICL ≥ 4; 5; 6; 7) and varying CFE-Mean. Table S2 shows the AUROC, the default and optimum threshold for CFE-Mean, alongside the sensitivity and specificity, for each curve. The default threshold for NavX (CFE-Mean ≤ 120 ms) always provided a high sensitivity, but poor specificity for CFAE detection. The proposed threshold (CFE-Mean ≤ 84 ms) provides the optimum sensitivity and specificity for CFAE detection in accordance to the CFAE classification performed by CARTO in all the cases.

The details of the ROC curves according to the CFAE classification performed by ICL (ICL ≥ 7) from the thirty set of 90% randomly selected AEGs are shown on Table S3. The details of the ROC curves according to the CFAE classification performed by CFE-Mean (CFE-Mean ≤ 120 ms) from the thirty set of 90% randomly selected AEGs are shown on Table S4.

**CFAE detection thresholds for CFE-StdDev, ACI and SCI**

***Methods***

The revised thresholds for both CFE-Mean and ICL found in the ROC curves were used concurrently to perform a new CFAE classification on the thirty sets of 697 randomly sampled AEGs. In this new classification, an AEG was classified as CFAE only if both CFE-Mean and ICL agreed with the classification using their revised thresholds. These new classifications were used to create ROC curves and hence obtain the optimum sensitivity and specificity thresholds for the complementary metrics – CFE-StdDev, ACI and SCI.

Therefore, the complementary metrics were used to identify AEGs that were classified as CFAEs by both CFE-Mean and ICL, using the revised thresholds.

***Results***

Table S5 shows the AUROC, the default and optimum threshold for CFE-StdDev, ACI and SCI, alongside the sensitivity and specificity, for each curve. The details of the Chi-square test and Cohen’s kappa for from the thirty set of 10% randomly selected AEGs for validation are shown on Table S6.

**Tables:**

**Table S1. Sensitivity and specificity for default and optimized ICL thresholds according CFE-Mean categorization.**

|  | ICL threshold | | Sensitivity | 1-Specificity | AUROC | P-value |
| --- | --- | --- | --- | --- | --- | --- |
| CFE-Mean ≤ 120 | Default | 7 | 0.492 | 0.05 | 0.854 | < 0.0001 |
|  | Optimum | 4 | 0.769 | 0.153 |  |  |
| CFE-Mean ≤ 110 | Default | 7 | 0.510 | 0.072 | 0.849 | < 0.0001 |
|  | Optimum | 4 | 0.790 | 0.191 |  |  |
| CFE-Mean ≤ 100 | Default | 7 | 0.527 | 0.093 | 0.829 | < 0.0001 |
|  | Optimum | 4 | 0.794 | 0.251 |  |  |
| CFE-Mean ≤ 90 | Default | 7 | 0.537 | 0.131 | 0.812 | < 0.0001 |
|  | Optimum | 4 | 0.804 | 0.301 |  |  |
| CFE-Mean ≤ 80 | Default | 7 | 0.547 | 0.175 | 0.793 | < 0.0001 |
|  | Optimum | 4 | 0.831 | 0.342 |  |  |
| CFE-Mean ≤ 70 | Default | 7 | 0.571 | 0.232 | 0.752 | < 0.0001 |
|  | Optimum | 5 | 0.760 | 0.357 |  |  |
| CFE-Mean ≤ 60 | Default | 7 | 0.550 | 0.292 | 0.701 | < 0.0001 |
|  | Optimum | 5 | 0.748 | 0.425 |  |  |
| CFE-Mean ≤ 50 | Default | 7 | 0.489 | 0.341 | 0.600 | 0.002 |
|  | Optimum | 4 | 0.761 | 0.560 |  |  |

AUROC = Area under receiver operating characteristic curve; ICL = Interval confidence level.

**Table S2. Sensitivity and specificity for default and optimized CFE-Mean thresholds according ICL categorization.**

|  | CFE-Mean  threshold (ms) | | Sensitivity | 1-Specificity | AUROC | P-value |
| --- | --- | --- | --- | --- | --- | --- |
| ICL ≥ 4 | Default | 120 | 0.920 | 0.387 | 0.823 | < 0.0001 |
|  | Optimum | 84.63 | 0.744 | 0.216 |  |  |
| ICL ≥ 5 | Default | 120 | 0.938 | 0.450 | 0.793 | < 0.0001 |
|  | Optimum | 84.18 | 0.765 | 0.270 |  |  |
| ICL ≥ 6 | Default | 120 | 0.948 | 0.502 | 0.765 |  |
|  | Optimum | 84.18 | 0.777 | 0.321 |  | < 0.0001 |
| ICL ≥ 7 | Default | 120 | 0.958 | 0.553 | 0.756 | < 0.0001 |
|  | Optimum | 84.18 | 0.807 | 0.361 |  |  |

AUROC = Area under receiver operating characteristic curve.

**Table S3. Sensitivity and specificity for default and optimized ICL thresholds according CFE-Mean categorization from the thirty set of 90% randomly selected AEGs.**

|  | ICL threshold (ms) | | Sensitivity | 1-Specificity | AUROC | P-value |
| --- | --- | --- | --- | --- | --- | --- |
| Test 1 | Default | 7 | 0.495 | 0.060 | 0.859 | P<0.0001 |
|  | Revised | 3 | 0.835 | 0.200 |  |  |
| Test 2 | Default | 7 | 0.499 | 0.043 | 0.858 | P<0.0001 |
|  | Revised | 3 | 0.830 | 0.216 |  |  |
| Test 3 | Default | 7 | 0.496 | 0.046 | 0.844 | P<0.0001 |
|  | Revised | 4 | 0.756 | 0.161 |  |  |
| Test 4 | Default | 7 | 0.506 | 0.047 | 0.855 | P<0.0001 |
|  | Revised | 4 | 0.772 | 0.156 |  |  |
| Test 5 | Default | 7 | 0.489 | 0.057 | 0.848 | P<0.0001 |
|  | Revised | 4 | 0.766 | 0.167 |  |  |
| Test 6 | Default | 7 | 0.485 | 0.052 | 0.851 | P<0.0001 |
|  | Revised | 4 | 0.761 | 0.146 |  |  |
| Test 7 | Default | 7 | 0.496 | 0.051 | 0.855 | P<0.0001 |
|  | Revised | 4 | 0.775 | 0.152 |  |  |
| Test 8 | Default | 7 | 0.497 | 0.048 | 0.861 | P<0.0001 |
|  | Revised | 4 | 0.768 | 0.138 |  |  |
| Test 9 | Default | 7 | 0.481 | 0.056 | 0.848 | P<0.0001 |
|  | Revised | 4 | 0.766 | 0.158 |  |  |
| Test 10 | Default | 7 | 0.491 | 0.046 | 0.853 | P<0.0001 |
|  | Revised | 4 | 0.765 | 0.144 |  |  |

**Table S3. Cont.**

|  | ICL threshold (ms) | | Sensitivity | 1-Specificity | AUROC | P-value |
| --- | --- | --- | --- | --- | --- | --- |
| Test 11 | Default | 7 | 0.481 | 0.047 | 0.850 | P<0.0001 |
|  | Revised | 4 | 0.764 | 0.160 |  |  |
| Test 12 | Default | 7 | 0.490 | 0.048 | 0.848 | P<0.0001 |
|  | Revised | 4 | 0.769 | 0.159 |  |  |
| Test 13 | Default | 7 | 0.499 | 0.049 | 0.858 | P<0.0001 |
|  | Revised | 3 | 0.828 | 0.211 |  |  |
| Test 14 | Default | 7 | 0.491 | 0.050 | 0.855 | P<0.0001 |
|  | Revised | 4 | 0.779 | 0.158 |  |  |
| Test 15 | Default | 7 | 0.501 | 0.052 | 0.851 | P<0.0001 |
|  | Revised | 4 | 0.767 | 0.151 |  |  |
| Test 16 | Default | 7 | 0.482 | 0.051 | 0.849 | P<0.0001 |
|  | Revised | 4 | 0.762 | 0.164 |  |  |
| Test 17 | Default | 7 | 0.499 | 0.053 | 0.849 | P<0.0001 |
|  | Revised | 4 | 0.768 | 0.170 |  |  |
| Test 18 | Default | 7 | 0.495 | 0.043 | 0.855 | P<0.0001 |
|  | Revised | 4 | 0.769 | 0.154 |  |  |
| Test 19 | Default | 7 | 0.479 | 0.044 | 0.852 | P<0.0001 |
|  | Revised | 3 | 0.817 | 0.206 |  |  |
| Test 20 | Default | 7 | 0.504 | 0.049 | 0.856 | P<0.0001 |
|  | Revised | 4 | 0.773 | 0.153 |  |  |

**Table S3. Cont.**

|  | ICL threshold (ms) | | Sensitivity | 1-Specificity | AUROC | P-value |
| --- | --- | --- | --- | --- | --- | --- |
| Test 21 | Default | 7 | 0.475 | 0.049 | 0.849 | P<0.0001 |
|  | Revised | 4 | 0.759 | 0.147 |  |  |
| Test 22 | Default | 7 | 0.503 | 0.043 | 0.856 | P<0.0001 |
|  | Revised | 4 | 0.771 | 0.159 |  |  |
| Test 23 | Default | 7 | 0.499 | 0.051 | 0.856 | P<0.0001 |
|  | Revised | 4 | 0.782 | 0.162 |  |  |
| Test 24 | Default | 7 | 0.499 | 0.046 | 0.852 | P<0.0001 |
|  | Revised | 4 | 0.762 | 0.147 |  |  |
| Test 25 | Default | 7 | 0.491 | 0.047 | 0.854 | P<0.0001 |
|  | Revised | 4 | 0.766 | 0.150 |  |  |
| Test 26 | Default | 7 | 0.493 | 0.051 | 0.850 | P<0.0001 |
|  | Revised | 4 | 0.770 | 0.164 |  |  |
| Test 27 | Default | 7 | 0.492 | 0.056 | 0.841 | P<0.0001 |
|  | Revised | 4 | 0.756 | 0.160 |  |  |
| Test 28 | Default | 7 | 0.478 | 0.057 | 0.844 | P<0.0001 |
|  | Revised | 3 | 0.812 | 0.217 |  |  |
| Test 29 | Default | 7 | 0.483 | 0.056 | 0.851 | P<0.0001 |
|  | Revised | 4 | 0.763 | 0.149 |  |  |
| Test 30 | Default | 7 | 0.500 | 0.038 | 0.863 | P<0.0001 |
|  | Revised | 4 | 0.768 | 0.139 |  |  |

**Table S4. Sensitivity and specificity for default and optimized ICL thresholds according CFE-Mean categorization from the thirty set of 90% randomly selected AEGs.**

|  | CFE-Mean  threshold (ms) | | Sensitivity | 1-Specificity | AUROC | P-value |
| --- | --- | --- | --- | --- | --- | --- |
| Test 1 | Default | 120 | 0.957 | 0.572 | 0.752 | P<0.0001 |
|  | Revised | 84.4 | 0.806 | 0.371 |  |  |
| Test 2 | Default | 120 | 0.964 | 0.552 | 0.753 | P<0.0001 |
|  | Revised | 84.4 | 0.822 | 0.374 |  |  |
| Test 3 | Default | 120 | 0.960 | 0.541 | 0.757 | P<0.0001 |
|  | Revised | 84.2 | 0.798 | 0.359 |  |  |
| Test 4 | Default | 120 | 0.961 | 0.546 | 0.757 | P<0.0001 |
|  | Revised | 84.4 | 0.813 | 0.351 |  |  |
| Test 5 | Default | 120 | 0.952 | 0.559 | 0.742 | P<0.0001 |
|  | Revised | 84.2 | 0.804 | 0.374 |  |  |
| Test 6 | Default | 120 | 0.955 | 0.557 | 0.747 | P<0.0001 |
|  | Revised | 84.2 | 0.801 | 0.359 |  |  |
| Test 7 | Default | 120 | 0.956 | 0.542 | 0.762 | P<0.0001 |
|  | Revised | 84.2 | 0.815 | 0.355 |  |  |
| Test 8 | Default | 120 | 0.960 | 0.553 | 0.750 | P<0.0001 |
|  | Revised | 84.2 | 0.806 | 0.366 |  |  |
| Test 9 | Default | 120 | 0.951 | 0.554 | 0.751 | P<0.0001 |
|  | Revised | 84.2 | 0.795 | 0.360 |  |  |
| Test 10 | Default | 120 | 0.959 | 0.545 | 0.752 | P<0.0001 |
|  | Revised | 82.1 | 0.780 | 0.361 |  |  |

**Table S4. Cont.**

|  | CFE-Mean  Threshold (ms) | | Sensitivity | 1-Specificity | AUROC | P-value |
| --- | --- | --- | --- | --- | --- | --- |
| Test 11 | Default | 120 | 0.959 | 0.555 | 0.755 | P<0.0001 |
|  | Revised | 84.2 | 0.807 | 0.363 |  |  |
| Test 12 | Default | 120 | 0.952 | 0.564 | 0.758 | P<0.0001 |
|  | Revised | 84.2 | 0.806 | 0.366 |  |  |
| Test 13 | Default | 120 | 0.961 | 0.526 | 0.750 | P<0.0001 |
|  | Revised | 84.2 | 0.801 | 0.367 |  |  |
| Test 14 | Default | 120 | 0.955 | 0.536 | 0.763 | P<0.0001 |
|  | Revised | 84.0 | 0.824 | 0.362 |  |  |
| Test 15 | Default | 120 | 0.957 | 0.549 | 0.760 | P<0.0001 |
|  | Revised | 84.2 | 0.815 | 0.354 |  |  |
| Test 16 | Default | 120 | 0.955 | 0.554 | 0.754 | P<0.0001 |
|  | Revised | 84.2 | 0.799 | 0.362 |  |  |
| Test 17 | Default | 120 | 0.961 | 0.558 | 0.753 | P<0.0001 |
|  | Revised | 84.2 | 0.805 | 0.365 |  |  |
| Test 18 | Default | 120 | 0.964 | 0.554 | 0.757 | P<0.0001 |
|  | Revised | 84.4 | 0.805 | 0.361 |  |  |
| Test 19 | Default | 120 | 0.963 | 0.571 | 0.748 | P<0.0001 |
|  | Revised | 84.2 | 0.800 | 0.372 |  |  |
| Test 20 | Default | 120 | 0.961 | 0.562 | 0.752 | P<0.0001 |
|  | Revised | 84.2 | 0.807 | 0.365 |  |  |

**Table S4. Cont.**

|  | CFE-Mean  threshold (ms) | | Sensitivity | 1-Specificity | AUROC | P-value |
| --- | --- | --- | --- | --- | --- | --- |
| Test 21 | Default | 120 | 0.959 | 0.574 | 0.748 | P<0.0001 |
|  | Revised | 84.2 | 0.803 | 0.373 |  |  |
| Test 22 | Default | 120 | 0.965 | 0.552 | 0.758 | P<0.0001 |
|  | Revised | 84.2 | 0.812 | 0.357 |  |  |
| Test 23 | Default | 120 | 0.956 | 0.543 | 0.762 | P<0.0001 |
|  | Revised | 84.4 | 0.825 | 0.365 |  |  |
| Test 24 | Default | 120 | 0.960 | 0.538 | 0.756 | P<0.0001 |
|  | Revised | 84.2 | 0.815 | 0.350 |  |  |
| Test 25 | Default | 120 | 0.960 | 0.549 | 0.762 | P<0.0001 |
|  | Revised | 84.2 | 0.810 | 0.356 |  |  |
| Test 26 | Default | 120 | 0.956 | 0.549 | 0.754 | P<0.0001 |
|  | Revised | 84.2 | 0.807 | 0.364 |  |  |
| Test 27 | Default | 120 | 0.952 | 0.553 | 0.757 | P<0.0001 |
|  | Revised | 84.2 | 0.808 | 0.356 |  |  |
| Test 28 | Default | 120 | 0.951 | 0.561 | 0.753 | P<0.0001 |
|  | Revised | 84.2 | 0.799 | 0.362 |  |  |
| Test 29 | Default | 120 | 0.951 | 0.553 | 0.750 | P<0.0001 |
|  | Revised | 84.2 | 0.792 | 0.361 |  |  |
| Test 30 | Default | 120 | 0.968 | 0.551 | 0.762 | P<0.0001 |
|  | Revised | 84.2 | 0.825 | 0.360 |  |  |

**Table S5. Sensitivity and specificity for CFE-StdDev, ACI and SCI ROC curves according to the CFAE classification in agreement between CFE-Mean and ICL.**

|  | Threshold (ms) | | Sensitivity | 1-Specificity | AUROC | P-value |
| --- | --- | --- | --- | --- | --- | --- |
| Test 1 | CFE-StdDev | 47.06 | 0.900 | 0.189 | 0.878 | P<0.0001 |
|  | ACI | 82.08 | 0.826 | 0.363 | 0.757 |  |
|  | SCI | 58.75 | 0.817 | 0.293 | 0.808 |  |
| Test 2 | CFE-StdDev | 47.73 | 0.922 | 0.202 | 0.873 | P<0.0001 |
|  | ACI | 82.92 | 0.846 | 0.366 | 0.757 |  |
|  | SCI | 58.75 | 0.814 | 0.304 | 0.810 |  |
| Test 3 | CFE-StdDev | 46.31 | 0.898 | 0.181 | 0.877 | P<0.0001 |
|  | ACI | 82.08 | 0.819 | 0.361 | 0.756 |  |
|  | SCI | 57.92 | 0.795 | 0.287 | 0.812 |  |
| Test 4 | CFE-StdDev | 45.73 | 0.896 | 0.183 | 0.876 | P<0.0001 |
|  | ACI | 82.92 | 0.853 | 0.379 | 0.753 |  |
|  | SCI | 58.75 | 0.833 | 0.307 | 0.812 |  |
| Test 5 | CFE-StdDev | 45.52 | 0.890 | 0.186 | 0.876 | P<0.0001 |
|  | ACI | 82.08 | 0.813 | 0.359 | 0.754 |  |
|  | SCI | 58.75 | 0.813 | 0.304 | 0.806 |  |
| Test 6 | CFE-StdDev | 45.73 | 0.891 | 0.181 | 0.876 | P<0.0001 |
|  | ACI | 82.08 | 0.827 | 0.372 | 0.747 |  |
|  | SCI | 58.75 | 0.823 | 0.313 | 0.805 |  |
| Test 7 | CFE-StdDev | 46.55 | 0.900 | 0.176 | 0.886 | P<0.0001 |
|  | ACI | 82.08 | 0.820 | 0.348 | 0.767 |  |
|  | SCI | 58.75 | 0.817 | 0.282 | 0.819 |  |

**Table S5. Cont.**

|  | Threshold (ms) | | Sensitivity | 1-Specificity | AUROC | P-value |
| --- | --- | --- | --- | --- | --- | --- |
| Test 8 | CFE-StdDev | 46.55 | 0.903 | 0.191 | 0.876 | P<0.0001 |
|  | ACI | 82.08 | 0.829 | 0.357 | 0.759 |  |
|  | SCI | 57.92 | 0.803 | 0.291 | 0.814 |  |
| Test 9 | CFE-StdDev | 45.71 | 0.891 | 0.178 | 0.877 | P<0.0001 |
|  | ACI | 82.08 | 0.819 | 0.354 | 0.757 |  |
|  | SCI | 58.75 | 0.805 | 0.304 | 0.805 |  |
| Test 10 | CFE-StdDev | 47.73 | 0.918 | 0.208 | 0.868 | P<0.0001 |
|  | ACI | 82.08 | 0.830 | 0.360 | 0.760 |  |
|  | SCI | 58.75 | 0.833 | 0.298 | 0.813 |  |
| Test 11 | CFE-StdDev | 46.55 | 0.902 | 0.187 | 0.875 | P<0.0001 |
|  | ACI | 82.08 | 0.821 | 0.364 | 0.756 |  |
|  | SCI | 58.75 | 0.828 | 0.314 | 0.804 |  |
| Test 12 | CFE-StdDev | 45.73 | 0.894 | 0.172 | 0.887 | P<0.0001 |
|  | ACI | 82.08 | 0.825 | 0.344 | 0.771 |  |
|  | SCI | 57.92 | 0.798 | 0.273 | 0.824 |  |
| Test 13 | CFE-StdDev | 47.73 | 0.921 | 0.199 | 0.879 | P<0.0001 |
|  | ACI | 82.08 | 0.823 | 0.362 | 0.759 |  |
|  | SCI | 57.92 | 0.797 | 0.298 | 0.808 |  |
| Test 14 | CFE-StdDev | 47.73 | 0.927 | 0.190 | 0.880 | P<0.0001 |
|  | ACI | 82.08 | 0.832 | 0.338 | 0.774 |  |
|  | SCI | 58.75 | 0.815 | 0.287 | 0.818 |  |

**Table S5. Cont.**

|  | Threshold (ms) | | Sensitivity | 1-Specificity | AUROC | P-value |
| --- | --- | --- | --- | --- | --- | --- |
| Test 15 | CFE-StdDev | 46.55 | 0.907 | 0.176 | 0.884 | P<0.0001 |
|  | ACI | 82.08 | 0.827 | 0.355 | 0.764 |  |
|  | SCI | 58.75 | 0.823 | 0.302 | 0.810 |  |
| Test 16 | CFE-StdDev | 47.73 | 0.922 | 0.184 | 0.890 | P<0.0001 |
|  | ACI | 82.08 | 0.823 | 0.360 | 0.761 |  |
|  | SCI | 58.75 | 0.823 | 0.305 | 0.810 |  |
| Test 17 | CFE-StdDev | 45.73 | 0.893 | 0.186 | 0.875 | P<0.0001 |
|  | ACI | 82.08 | 0.830 | 0.365 | 0.759 |  |
|  | SCI | 57.92 | 0.807 | 0.297 | 0.811 |  |
| Test 18 | CFE-StdDev | 47.73 | 0.930 | 0.186 | 0.883 | P<0.0001 |
|  | ACI | 82.92 | 0.850 | 0.370 | 0.765 |  |
|  | SCI | 58.75 | 0.823 | 0.302 | 0.813 |  |
| Test 19 | CFE-StdDev | 45.52 | 0.892 | 0.180 | 0.878 | P<0.0001 |
|  | ACI | 82.92 | 0.842 | 0.370 | 0.756 |  |
|  | SCI | 58.75 | 0.815 | 0.305 | 0.810 |  |
| Test 20 | CFE-StdDev | 45.71 | 0.898 | 0.170 | 0.882 | P<0.0001 |
|  | ACI | 82.08 | 0.832 | 0.353 | 0.762 |  |
|  | SCI | 58.75 | 0.815 | 0.315 | 0.810 |  |
| Test 21 | CFE-StdDev | 46.09 | 0.896 | 0.185 | 0.878 | P<0.0001 |
|  | ACI | 82.08 | 0.818 | 0.358 | 0.754 |  |
|  | SCI | 58.75 | 0.815 | 0.303 | 0.810 |  |

**Table S5. Cont.**

|  | Threshold (ms) | | Sensitivity | 1-Specificity | AUROC | P-value |
| --- | --- | --- | --- | --- | --- | --- |
| Test 22 | CFE-StdDev | 45.73 | 0.897 | 0.176 | 0.880 | P<0.0001 |
|  | ACI | 82.08 | 0.830 | 0.363 | 0.759 |  |
|  | SCI | 57.92 | 0.807 | 0.292 | 0.814 |  |
| Test 23 | CFE-StdDev | 47.85 | 0.925 | 0.192 | 0.882 | P<0.0001 |
|  | ACI | 82.08 | 0.818 | 0.344 | 0.766 |  |
|  | SCI | 58.75 | 0.821 | 0.305 | 0.817 |  |
| Test 24 | CFE-StdDev | 46.55 | 0.902 | 0.185 | 0.875 | P<0.0001 |
|  | ACI | 82.08 | 0.818 | 0.345 | 0.762 |  |
|  | SCI | 59.58 | 0.842 | 0.305 | 0.824 |  |
| Test 25 | CFE-StdDev | 46.09 | 0.899 | 0.182 | 0.879 | P<0.0001 |
|  | ACI | 82.92 | 0.845 | 0.367 | 0.758 |  |
|  | SCI | 58.75 | 0.814 | 0.302 | 0.810 |  |
| Test 26 | CFE-StdDev | 47.20 | 0.913 | 0.186 | 0.877 | P<0.0001 |
|  | ACI | 82.08 | 0.820 | 0.358 | 0.757 |  |
|  | SCI | 58.75 | 0.823 | 0.310 | 0.807 |  |
| Test 27 | CFE-StdDev | 47.06 | 0.902 | 0.179 | 0.889 | P<0.0001 |
|  | ACI | 82.08 | 0.824 | 0.366 | 0.763 |  |
|  | SCI | 58.75 | 0.837 | 0.299 | 0.819 |  |
| Test 28 | CFE-StdDev | 47.20 | 0.911 | 0.186 | 0.880 | P<0.0001 |
|  | ACI | 82.08 | 0.819 | 0.364 | 0.753 |  |
|  | SCI | 58.75 | 0.826 | 0.304 | 0.808 |  |

**Table S5. Cont.**

|  | Threshold (ms) | | Sensitivity | 1-Specificity | AUROC | P-value |
| --- | --- | --- | --- | --- | --- | --- |
| Test 29 | CFE-StdDev | 45.52 | 0.883 | 0.177 | 0.874 | P<0.0001 |
|  | ACI | 82.08 | 0.811 | 0.365 | 0.751 |  |
|  | SCI | 58.75 | 0.801 | 0.296 | 0.811 |  |
| Test 30 | CFE-StdDev | 46.55 | 0.914 | 0.192 | 0.808 | P<0.0001 |
|  | ACI | 82.08 | 0.825 | 0.367 | 0.749 |  |
|  | SCI | 57.92 | 0.801 | 0.291 | 0.808 |  |

**Table S6. Chi-square test and Cohen’s kappa CFAE classification performed by default and optimized thresholds for NavX and CARTO from the validation dataset.**

|  | Thresholds | χ² | P-values | Kappa | P-values |
| --- | --- | --- | --- | --- | --- |
| Test 1 | Default | 24.5 | <0.0001 | 0.423 | <0.0001 |
|  | Revised | 13.9 |  | 0.387 |  |
| Test 2 | Default | 11.2 | 0.001 | 0.283 | 0.001 |
|  | Revised | 21.1 | <0.0001 | 0.473 | <0.0001 |
| Test 3 | Default | 10.4 | 0.001 | 0.257 | 0.001 |
|  | Revised | 14.8 | <0.0001 | 0.402 | <0.0001 |
| Test 4 | Default | 9.6 | 0.002 | 0.241 | 0.001 |
|  | Revised | 21.7 | <0.0001 | 0.486 | <0.0001 |
| Test 5 | Default | 23.1 | <0.0001 | 0.404 | <0.0001 |
|  | Revised | 34.8 |  | 0.609 |  |
| Test 6 | Default | 20.8 | <0.0001 | 0.393 | <0.0001 |
|  | Revised | 28.9 |  | 0.556 |  |
| Test 7 | Default | 13.0 | <0.0001 | 0.281 | <0.0001 |
|  | Revised | 15.9 |  | 0.419 |  |
| Test 8 | Default | 13.5 | <0.0001 | 0.305 | <0.0001 |
|  | Revised | 15.2 |  | 0.408 |  |
| Test 9 | Default | 23.4 | <0.0001 | 0.409 | <0.0001 |
|  | Revised | 21.2 |  | 0.480 |  |
| Test 10 | Default | 12.8 | <0.0001 | 0.295 | <0.0001 |
|  | Revised | 19.6 |  | 0.458 |  |

**Table S8. Cont.**

|  | Thresholds | χ² | P-values | Kappa | P-values |
| --- | --- | --- | --- | --- | --- |
| Test 11 | Default | 18.7 | <0.0001 | 0.382 | <0.0001 |
|  | Revised | 25.8 |  | 0.518 |  |
| Test 12 | Default | 24.3 | <0.0001 | 0.419 | <0.0001 |
|  | Revised | 9.1 | 0.003 | 0.321 | 0.001 |
| Test 13 | Default | 15.0 | <0.0001 | 0.328 | <0.0001 |
|  | Revised | 21.1 |  | 0.475 |  |
| Test 14 | Default | 11.6 | 0.001 | 0.260 | <0.0001 |
|  | Revised | 11.9 |  | 0.366 |  |
| Test 15 | Default | 13.5 | <0.0001 | 0.288 | <0.0001 |
|  | Revised | 18.5 |  | 0.451 |  |
| Test 16 | Default | 20.4 | <0.0001 | 0.388 | <0.0001 |
|  | Revised | 10.7 | 0.001 | 0.343 | 0.001 |
| Test 17 | Default | 16.8 | <0.0001 | 0.338 | <0.0001 |
|  | Revised | 29.1 |  | 0.554 |  |
| Test 18 | Default | 12.9 | <0.0001 | 0.311 | <0.0001 |
|  | Revised | 9.7 | 0.002 | 0.330 | 0.001 |
| Test 19 | Default | 24.2 | <0.0001 | 0.466 | <0.0001 |
|  | Revised | 43.0 |  | 0.674 |  |
| Test 20 | Default | 12.8 | <0.0001 | 0.295 | <0.0001 |
|  | Revised | 18.7 |  | 0.454 |  |

**Table S8. Cont.**

|  | Thresholds | χ² | P-values | Kappa | P-values |
| --- | --- | --- | --- | --- | --- |
| Test 21 | Default | 30.0 | <0.0001 | 0.521 | <0.0001 |
|  | Revised | 17.4 |  | 0.437 |  |
| Test 22 | Default | 9.5 | 0.002 | 0.255 | 0.001 |
|  | Revised | 19.7 | <0.0001 | 0.463 | <0.0001 |
| Test 23 | Default | 12.5 | <0.0001 | 0.272 | <0.0001 |
|  | Revised | 18.9 |  | 0.454 |  |
| Test 24 | Default | 9.0 | 0.003 | 0.232 | 0.001 |
|  | Revised | 5.8 | 0.016 | 0.261 | 0.009 |
| Test 25 | Default | 14.0 | <0.0001 | 0.313 | <0.0001 |
|  | Revised | 21.1 |  | 0.480 |  |
| Test 26 | Default | 15.8 | <0.0001 | 0.323 | <0.0001 |
|  | Revised | 16.9 |  | 0.430 |  |
| Test 27 | Default | 19.9 | <0.0001 | 0.361 | <0.0001 |
|  | Revised | 16.7 |  | 0.422 |  |
| Test 28 | Default | 27.4 | <0.0001 | 0.459 | <0.0001 |
|  | Revised | 25.0 |  | 0.519 |  |
| Test 29 | Default | 22.4 | <0.0001 | 0.396 | <0.0001 |
|  | Revised | 43.8 |  | 0.680 |  |
| Test 30 | Default | 8.4 | 0.004 | 0.247 | 0.002 |
|  | Revised | 9.9 | 0.002 | 0.334 | 0.001 |

**Figure S1**


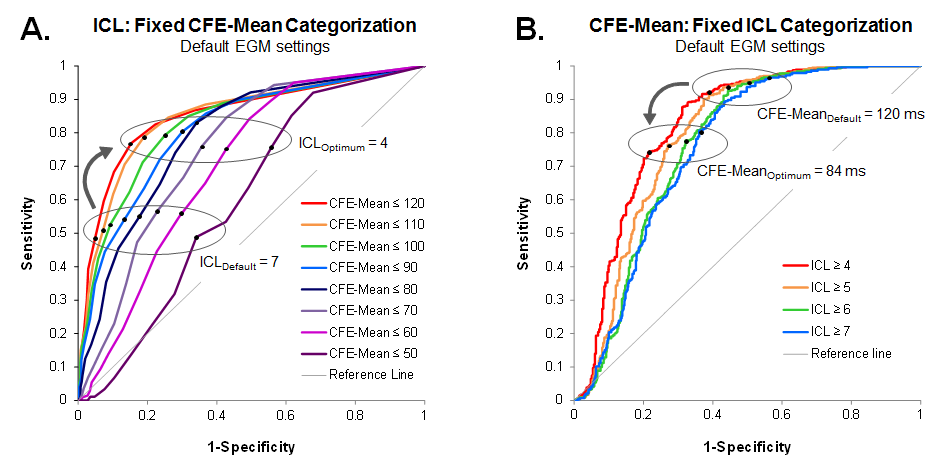


**Figure S2.** Receiver operating characteristic (ROC) curves based on ICL or CFE-Mean categorization. ROC curves of **A.** ICL according to CFAE classification using fixed CFE-Mean thresholds (CFE-Mean ≤ 120; 110; 100; 90; 80; 70; 60; 50 ms) and; **B.** CFE-Mean according to CFAE classification using fixed ICL thresholds (ICL ≥ 4; 5; 6; 7).
